# Knowledge, Expertise and Science Advice During COVID-19: In Search of Epistemic Justice for the ‘Wicked’ Problems of Post-Normal Times

**DOI:** 10.1080/02691728.2022.2103750

**Published:** 2022-10-10

**Authors:** Maru Mormina

**Affiliations:** Ethox Centre and Wellcome Centre for Ethics and Humanities, Big Data Institute, Li Ka Shing Centre for Health Information and Discovery, University of Oxford, Oxford, UK

**Keywords:** Expertise, COVID-19, epistemic injustice, post-normal science

## Abstract

A consistent claim from governments around the world during the Coronavirus pandemic has been that they were *following the science*. This raises the question, central to this paper, of what and whose knowledge is or should be sought, which is being side-lined through the choice of particular framings and discourses, and with what consequences for the creation and implementation of evidence-based policy to tackle wicked problems. Through the lens of Fricker’s epistemic injustice, I problematise the expertise that has guided the COVID-19 response as epistemically narrow and argue that counteracting a monolithic culture of expertise requires tackling the structural inequalities in the systems of knowledge production to diversify the social and epistemological foundations of science. Drawing on Post-normal Science (PNS) theory, I suggest that the expertise needed to respond to the challenges of a post-COVID world is one that embraces greater pluralism, avoids groupthink, challenges the accepted orthodoxy and helps us revert old models and rigid path dependencies that so often neglect the lived realities and demands of those left behind. This can only be realised by overcoming epistemic injustice and embracing epistemic democracy in the practice of evidence-based policy.

## Introduction

The starting premise of this paper is that whilst COVID-19 is one of the greatest global crises in recent history, it is not a single isolated event. It is a complex interlocking of mutually reinforcing socioeconomic and environmental variables – a wicked problem (Rittel and Webber 1973), requiring social, economic, and environmental, as well as medical solutions. This raises the question, central to this paper, of what and whose knowledge is or should be sought, which is being side-lined through the choice of particular framings and discourses, and with what consequences for the creation and implementation of ‘evidence-based policy’ (EBP) during a pandemic and its aftermath.

The paper problematizes the narrow use of expertise during COVID-19. It shows through examples that COVID-19 has largely been framed as a health emergency instead of a complex ‘wicked’ problem. This has constrained the expertise mobilised in the policy response. The narrow use of expertise is considered as an epistemic injustice (Fricker 2007) against certain types of knowledge and epistemic agents, which, at the height of the pandemic, created credibility and intelligibility deficits within the institutions of science advice. This foreclosed policy options and precluded an understanding of the impacts of policies upon the most disadvantaged in society. Following Anderson (2012), the paper traces back these epistemic injustices to historical structural inequalities in the institutions of knowledge. It argues that pandemics, like all wicked problems, demand holistic approaches to EBP and this cannot be done without disrupting a monolithic culture of expertise at its roots, ensuring broad participation in knowledge production and fostering epistemic diversity in science advisory bodies. But this is not enough. Crises bearing the hallmark of wicked problems are situations where ‘*facts are uncertain, values in dispute, stakes high and decisions urgent’* (Ravetz 1999). Under these conditions, expert advice is challenged by scientific uncertainties, and conflicting values allow for conflicting interpretations of evidence, public criticism and contestation of decision-making process. For this reason, the paper looks at the COVID-19 pandemic through the lens of post-normal science (PNS) (Funtowicz and Ravetz 1993) to argue for a policy response grounded in a much deeper engagement with the values of deliberative democracy. This means not only ensuring epistemic pluralism in expert advisory groups but above all epistemic pluralism in the decision-making process, by bringing to the table the knowledge extant in those invested in the problem, especially groups traditionally excluded from deliberative and decision-making fora.

The increased reliance on scientific experts has been one of the most salient features of the COVID-19 pandemic. To build trust, reassure the public, and justify sometimes draconian policies, governments across the world have claimed to ‘follow the science’. The *science*, however, has been uncertain and contested and yet, repeatedly called to mediate in politically charged questions. In many cases, this meant the views of a relatively small – and arguably homogenous – group of largely insider advisors (Cairney 2021) often asked to act as neutral honest brokers (Pielke 2007), a task difficult to achieve under conditions of uncertainty when judgement is required but it becomes inevitably intertwined with worldviews, values and interests. For this reason, it is important to ask *whose* values are providing the bridge between evidence and decision-making, since different value configurations may lead to different ways to appraise and communicate such evidence. Much of public scrutiny of science advice focuses on the *credibility* of the data, but there is little debate on the *values* informing the interpretation of the data and models that have weighed so heavily in the critical policy decisions made during the pandemic.

Whilst acknowledging that the use of evidence and expert advice in policy making is a political process, this paper focuses neither on the process nor on the politics. The paper does not focus so much on *how* or *whether* policymakers use scientific knowledge but, rather, on *why* only certain experts and types of knowledge influence policy. Building on a vast literature on expertise from both social epistemology and Science and Technology Studies, and drawing on specific examples, mostly from the UK, the paper critically examines *epistemic narrowing* within systems of science advice. The concept is used to describe the homogenisation of *what* is considered knowledge (often defined through technoscientific paradigms) and of *who* is considered an expert. Epistemic narrowing is used as a frame of reference against the pluralist ideal of participatory democracy to scrutinise the role of science advice in the context of the COVID-19 crisis.

Through the analytical lens of (super) wicked problem (Levin et al. 2012; Rittel and Webber 1973), the paper demonstrates how the framing of COVID-19 as a largely biomedical crisis may explain the narrow expertise mobilised through science advisory bodies the world over (Grundmann 2021; Priola and Pecis 2020; Rajan et al. 2020; Singh 2020; Wenham and Herten-Crabb 2021). This matters epistemologically: besides the obvious exclusion of talent, non-participatory processes of science advice make it more difficult to escape scientific bias. It precludes the cognitive diversity needed for the governance of complex wicked problems such as pandemics. It also diminishes the epistemic character of advisory groups. Diverse groups not only have a greater repertoire of cognitive tools to perform knowledge-intensive tasks (Page 2017), but also the collective epistemic virtues (epistemic conscientiousness, open-mindedness, etc.) necessary in situations where thoughtful deliberation and shared understanding are key (Kim 2022).

However, epistemic narrowing in policymaking also matters morally, for it constitutes an epistemic injustice (Fricker 2007) manifested in the persistent side-lining of relevant forms of knowledge and experts. This can create policy blind spots, especially to the needs of the most disadvantaged. The idea of epistemic injustice has been widely used in the context of interpersonal relationships, but despite its relevance to policymaking, it has not been widely applied to EBP. Epistemic injustice provides a useful lens to dissect issues of pluralism and representation in the use of knowledge and evidence, specifically during the pandemic. Epistemic justice theory, however, focuses on the discriminatory aspects of epistemic injustice but it is more limited in its distributional requirements. In other words, epistemic justice can be understood as a negative obligation to refrain from epistemic prejudice but it does not necessarily impose a positive obligation to distribute epistemic power by giving different epistemic agents a seat at the decision-making table. Therefore, the paper combines a requirement of justice with a requirement of inclusion by drawing insights from PNS theory (Funtowicz and Ravetz 1993). PNS, however, entails a normative requirement for epistemic diversity and inclusive engagement in decision-making processes but it remains limited in its justice requirements to ensure that inclusive engagement is also meaningful engagement. Both theories, thus, are used in complement to offer some signposts that can help re-orient EBP in the post-COVID era.

## COVID-19: A Health Crisis or a Wicked Problem?

To understand the global management of the current pandemic it is useful to consider the challenges of social policy and public administration. It is widely acknowledged that policy problems are rarely solved. Quoting Wildavsky (1979, 386), *‘problems are not so much solved as alleviated, superseded, transformed, and otherwise dropped from view’*. Despite this, problem-solving remains an appealing imagery in public policy, nowhere more powerful than during crises.

Complex problems in social policy (from crime to poverty to pandemics) are inherently ill-structured and defy definition, i.e. even their definition and scope is contested. They are *wicked problems* (Rittel and Webber 1973) that cannot be easily solved, or even defined, due to uncertainty and incomplete knowledge. Instead, multiple and often incompatible characterisations are possible because the actors involved operate in different sectors and/or at different levels, and therefore see different aspects of the problem and become invested in different solutions. Wicked problems are not a single problem but a web of problems intersecting in complex ways as a largely unmanageable system. Such problems are not amenable to the conventional logical problem-solving approaches typically applied to linear or *tame* problems. Applying such approaches to wicked problems not only fails to tackle them but may create undesirable consequences.

The COVID-19 crisis has been widely described as a wicked problem (Angeli, Camporesi, and Dal Fabbro 2021; Lilleker and Stoeckle 2021; Rainey et al. 2021). What was the policy problem in the spring of 2020: saving lives, preventing the collapse of health systems, avoiding economic meltdown, all three, or something else altogether? Since there is no single definition there can be no single answer but a variety of multiple and often contradictory solutions. Lack of understanding about the virus’ mode of transmission translated into confusing definitions – ranging from flu-like comparisons to novel disease classifications – and wide variation among countries in policy responses (Ritchie et al. 2020), including the controversial pursuit of herd immunity (Horton 2020).

Some have argued that the pandemic represents a particularly wicked type of problems: a *super-wicked problem* (Auld et al. 2021; Levin et al. 2012; Schiefloe 2021), characterised by four features: those who must solve the problem are also causing it (we benefit from the very collective patterns of behaviour that contribute to the spread of the virus), there is no central authority (for example, to ensure coordinated international action), there is time pressure (a critical window of opportunity to tackle the problem), and long-term solutions are discounted due to their short-term costs. The last two features are particularly relevant to the present discussion.

### Time Pressure

Paradoxically, wicked problems are unsolvable, yet they demand action. For super-wicked problems, such action is particularly urgent. Framing COVID-19 as a health crisis has allowed decision makers to quickly direct the policy response towards the immediate threats: reduce the rate of transmission, avoid deaths and ensure that health systems are able to meet demand. In other words, action has been possible because a (super) wicked problem has been *tamed*, that is, constrained within certain boundaries and made amenable to linear problem-solving approaches. Such simplification resulted in delegation of responsibilities to a few actors – those whose expertise is perceived as relevant within the problem boundaries. In many countries, overall responsibility for pandemic response was transferred to health authorities (DHSC 2020; Wolf-Fordham 2020). For this reason, expertise was mostly drawn from the biomedical community (Rajan et al. 2020). Problem complexity was, in fact, traded off for simplification to effect swift action and establish lines of accountability, but it resulted in a tunnel vision and particular epistemic commitments to the specific framings (and solutions) within the purview of the narrow group of experts involved. This may underlie the scientific biases that initially underestimated the difference between COVID-19 and flu, and led to a pandemic response based on influenza modelling, the dominant form of expertise available within institutions with recognised epistemic authority in health, such as the WHO. It may also explain the conspicuous lack of attention to the complexity, interdependencies and ramifications of the measures advocated. A clear example were work from home and school closure mandates, advised (and imposed) without attention to the incompatibility of these demands for parents, especially women (Wenham and Herten-Crabb 2021).

### Discounting Long-Term Solutions

According to Baubion (2013), wicked problems are rapidly changing situations requiring networked responses that can quickly adapt and innovate. Such responses are often only possible with a ‘whole-of-government approach’ (WGA). WGA can be difficult to implement, especially within systems of siloed structures and under time pressure. Overall, countries that had better outcomes during the initial phase of the pandemic^1^ (e.g. Singapore, Uruguay, Taiwan) have generally displayed well-coordinated WGA responses compared to countries with poor outcomes (e.g. UK, US, Peru) (Haldane et al. 2021), suggesting that the former may have had pre-existing structures to swiftly bring together different parts of government to achieve a holistic, yet, rapid response.

Crucially, a WGA is critical to build system resilience going forward (Haldane et al. 2021). By narrowly framing COVID-19 as an unprecedented *health* situation, many governments have prioritised a siloed management of the pandemic and focused on short-term medical solutions, at the same time discounting long-term holistic approaches necessary to prepare health systems for future shocks. In the UK, this short-termism is reflected in the setup of bespoke management arrangements (for example, for test and trace) outside the usual government structures (such as the National Health Service), which can be rolled back once the threat is removed and *business as usual* resumed. Short-termism and lack of willingness to confront the long-term changes required to avert future crises continues to determine policy direction as societies emerge from the pandemic. After two years of pandemic response, we see globally a doubling down of the status quo, facilitated by an emphasis on technoscientific solutions, particularly vaccines. Undoubtedly, these are important to re-introduce some form of pre-pandemic normalcy, but they do not fully vindicate the narrow biomedical framing of the crisis. Vaccine-escape and waning immunity can make the arms race against new variants of concern hard to win, suggesting that biomedical solutions, whilst necessary, alone may not suffice. COVID-19 may have a biological origin in a non-human virus that jumped species but its development into an unprecedented worldwide pandemic responds to much deeper systemic weaknesses and vulnerabilities – bio-social, political, economic (Hulme et al. 2020; Smith 2021) – that the narrow framing of the crisis has hidden from view. Addressing these vulnerabilities is essential to build preparedness and resilience going forward but this requires other normative approaches and kinds of expertise.

## Science Advice and the COVID-19 Response

The section above argued that the narrow framing of the pandemic as a health crisis and failure to understand it as a wicked problem hampered the ability to fully tackle the problem in all its complexity and build system resilience. Such narrow framing may also explain the excessive reliance on narrow (largely biomedical) expertise and a focus on short-term medical solutions. However, to fully understand the narrowing of expertise during the COVID-19 response, we need to consider wider questions of epistemic diversity in science advice that determine whose knowledge counts, who is recognised as an expert and who is excluded.

Defining expertise is by no means a recent concern. Since Plato, different traditions of sociologists and philosophers have engaged with the question of how to recognise expertise. Goldman (2001, 2018) rejects a formulation of expertise based on social recognition, instead arguing for a substantive account of expertise, whilst also acknowledging that the fluidity of the concept means that different definitional criteria can be used in different contexts. Whilst substantive definitions are elusive, social epistemological accounts are not universally accepted, either, even though the relational dimension of expertise cannot be denied. Collins and Evans (2002) recognise that any definition of expertise ultimately must help distinguish experts from non-experts if it is to avoid what they call ‘the problem of extension’. In other words, to be an expert is not (only) a question of fulfilling a given number of criteria to demonstrate competence and experience but to stand in relation with someone else, a non-expert.

The aim of this paper is not to settle on an account of expertise but to use the context of the COVID-19 crisis to understand why certain experts and forms of expertise become dominant in government advisory processes, and with what consequences for the pursuit of equitable policies. The question, therefore, is not so much who is/should be an expert but how and why some experts acquire *recognition* within policy circles at the *exclusion* of other (equally or even more qualified) experts. Given this framing and the paper’s emphasis on one particular type of expert, the science advisor, zooming into the relational dimension of expertise through a social epistemological framework seems appropriate. Grundmann’s (2017) relational account of expertise is particularly suitable to explore how expertise is established, legitimised, and how it relates to epistemic narrowing by becoming the product and preserve of certain social groups. Grundman’s position can be summarised as follows: to be an expert implies that others are non-experts *(exclusion)*, and their standing depends on their expertise being acknowledged and demanded *(recognition).*

As a relationship of *exclusion*, expertise implies a knowledge differential that often runs alongside other social differentials: power, privilege, and prestige (Evetts, Mieg, and Felt 2006). For example, unequal distribution of government funding enable some institutions (and the individuals working for them) to build scientific standing at the exclusion of others. These differentials tend to be exacerbated over time, resulting in strong *Matthew effects*^2^ that benefit well-established institutions. In the UK, performance-based formulas for funding allocation benefit disproportionately institutions in the area known as the Golden Triangle (London and the Southeast). With a smaller share of funding, less established universities elsewhere have reduced opportunities to develop as centres of scientific excellence. As a result, governments relying on indicators of excellence to identify the best available expertise will inevitably draw from a relatively small pool of talent concentrated in a handful of selective institutions. An example of this phenomenon may be the composition of the UK Science Advisory Group for Emergencies (SAGE), a body convened on an ad-hoc basis to provide scientific advice to Prime Ministers during emergencies. Activated in early 2020, the group had an initial membership drawn mostly from a handful of elite universities, largely in the Golden Triangle.^3^

Readers may contend that if the goal is to base decisions on the best available science, it should not matter where the science comes from. However, if we accept that the governance of wicked problems raises the need to incorporate potentially conflicting or incommensurable types of knowledge and perspectives from diverse and often geographically dispersed sources, diversity does matter. Elite institutions often struggle to attract diverse staff (Nwonka 2019; White-Lewis 2020) and hence tend to be cognitively more homogeneous, which in turn impacts on the kind of expertise they make available to policymakers. It may also be argued that in stratified societies where epistemic authority is associated with established institutions, drawing expertise from these institutions can help legitimise scientific advice. This may be true in societies where individual meritocracy ties expert legitimacy to professional standing, but it certainly is not universal (Jasanoff 2005). Furthermore, recent history of anti-establishment feeling – from Trumpism to Brexit and more recently anti-vaccination and anti-lockdown movements – shows the limitations of exclusionary and elitist forms of expert legitimation (Grundmann 2018).

As a relationship of *recognition*, expertise, and particularly the expertise of a science advisor, implies some form of social esteem. Personal recommendation via established networks (peer recognition) or media presence (public recognition) are among the primary routes by which policymakers identify potential science advisors (Haynes et al. 2012). Without these ‘*more or less institutionalized relationships of mutual acquaintance and recognition*’ (Bourdieu and Wacquant 1992), individuals may be experts by some substantive account of expertise, but may not become advisors. This form of social capital that policy makers often take as a proxy for expertise is linked to class and other forms of social stratification *(ibid)*. Indeed, because it is usually easier to recognise and trust those with views and other characteristics similar to one’s own, selective use and commissioning of expert advice to support particular agendas (Katz and Matter 2017; Stevens 2007) or the exclusion of advisors whose views do not ‘fit’ (Stevens 2020) is commonplace. As a result, drawing experts from trusted networks of well-connected and like-minded individuals, rather than seeking a spectrum of views and diversity of experience, maintains a vicious circle of influence and narrow expertise.

The expertise that governments seek when appointing advisors may thus be less meritocratic and more ideological (Cairney 2021), and not infrequently that which is perceived to lend credibility to a pre-conceived policy agenda (Swank 2000). To illustrate this, consider how tensions within the UK government regarding the second wave of COVID-19 infections in late 2020 were resolved by the Prime Minister following a secret meeting with a group of elite health experts advocating the controversial ‘focused protection’ approach (Kulldorff, Gupta, and Bhattacharya 2020). This was a fringe view (although see Ioannidis 2022) promoting a more or less laissez-faire management of the pandemic to allow people greater freedom to pursue social and economic activity. Faced with competing demands from ministers advocating tougher public health measures and those lobbying for looser restrictions to protect the economy, the Prime Minister is said to have bypassed the official scientific advice (Calvert et al. 2020), siding instead with experts whose views seemed more consistent with his own alleged libertarian instincts (Tominey 2020). Whilst this could arguably represent a widening of science advice by bringing into the discussion alternative voices, the opaqueness surrounding the event rather suggests this may be an example of evidence selection and expert recognition based on ideology that can ultimately narrow the range of views feeding into the policy process.

## Epistemic Narrowing, Epistemic Injustice and the Dismissal of Needs During COVID-19

In EBP, the pronouncements of scientific advisors matter, at best, to guide the policy process, or at worst to provide post-hoc legitimacy (policy-based evidence) to decisions potentially affecting vast numbers of people. Epistemic narrowing in public policy is, therefore, not morally neutral. To better understand this moral dimension, the concept of *epistemic injustice* may offer a useful lens. Epistemic injustices occur when ‘*someone is wronged specifically in her capacity as a knower’* (Fricker 2007, 20), either because their knowledge or experience lacks credibility or because *‘socioepistemic structures’* (Dotson 2012) converge to marginalise them and exclude them from the process of knowledge production. In Fricker’s account, the former is described as *testimonial injustice* and the latter as *hermeneutical injustice*. An example of testimonial injustice is a witness (or in our case, an expert) whose testimony (or expertise) is deemed less credible by virtue of their gender, race, sexuality or other identity trait considered epistemically inferior. Hermeneutical injustices occur when someone’s experiences are not understood or rationalised due to their historic exclusion from the structures and activities that shape knowledge creation. A classic example is sexual harassment: whilst in the 21^st^ century, campaigns such as #Me Too have helped articulate its meaning, the same experience would have been unintelligible to previous generations due to the systematic exclusion of women from processes of collective sensemaking.

Hermeneutical injustices are therefore structural forms of injustices concerning the rules governing the relationships between individuals in their capacity as epistemic agents that cause them to be excluded from the systems and institutions of knowledge. Testimonial injustices are mostly transactional injustices relating to individual exchanges between agents, though some exchanges may be unfair due to background structural injustices (Anderson 2012). Testimonial injustice results in credibility deficits of certain individuals/groups and the concomitant credibility excess of others. Hermeneutical injustice, on the other hand, creates intelligibility deficits. Credibility and intelligibility deficits affect people status as epistemic agents thereby diminishing their social power, that is, their capacity ‘*to influence how things go in the social world*’ (Fricker 2007, 9). These deficits often run alongside other forms of disadvantage and therefore harm most directly those whose interests and voices are the least powerful.

Fricker’s account of epistemic injustice makes apparent the direct relationship between the ability of individuals to meaningfully contribute to public life (including policy making) and their credibility and intelligibility. The concept, therefore, seems particularly relevant to the normative questions arising from the use of expert advice in public policy. Whose knowledge counts in policymaking and why? Why should it matter that some types of knowledge and knowers are not recognised as valid contributions to public policy? These questions relate not so much to the actions of individual agents in the policy process, but to the structures and systems of knowledge production and mobilisation, and the rules that govern epistemic relationships between policy makers and their advisors. When seen through the lens of epistemic injustice, epistemic narrowing in public policy intersects with broader discourses of inequality, power and discrimination.

The use of expertise in public policy during COVID-19 raises important issues at the intersection of epistemology, ethics and politics, and for this reason the notion of epistemic injustice is a useful conceptual tool to bridge these three domains. To the question of why there have been policy failures in almost every country while trying to respond to the pandemic, answers can range from genuine scientific (epistemological) uncertainty regarding a novel virus, to (political) conflicts between different interests, to (moral) trade-offs between the needs of public health and those of the public purse. In all these domains, the policy process has privileged particular types of science and science experts. It has further cemented historic epistemic biases towards privileged expert elites with high social capital and towards forms of knowledge and approaches rooted in a Cartesian reductionism that have become associated with expertise but are far from being the only way.

Thus, while some policy failures may have been inevitable given the complex, uncertain and unprecedented situation, others expose pre-existing epistemic biases and injustices within the current systems of knowledge. For example, the behavioural assumptions made by the UK government’s Behavioural Insight Team, which applies nudge theory to public policy, wrongly predicted poor population compliance with strict and protracted quarantine measures, against decades of public health knowledge suggesting the opposite. These predictions led to the delayed government response during the critical early phases of the pandemic (Hunter 2020), costing thousands of avoidable deaths. This narrow use of disciplinary expertise was a testimonial injustice involving a credibility excess of reductionist behavioural sciences and a credibility deficit of more holistic public health expertise. Around the world, expert task forces and decision-making bodies have been largely male dominated (van Daalen et al. 2020). Gender bias may explain why certain policies amplified pre-existing gender-based inequities. For example, lockdowns mandates exacerbated women’s pre-existing economic precarity, as their traditional role as main caretakers within the family were not fully accounted, resulting in increased caring responsibilities and higher levels of income loss compared to men (Wenham and Herten-Crabb 2021). Failure to account for intersectional needs constitutes a form of hermeneutical injustice linked to intelligibility deficits of women, whose lived realities were not adequately represented within policy communities.

Epistemic injustices during Covid-19 extend beyond the credibility deficit of traditional public health or the intelligibility deficits of women. The announcement of India’s nationwide lockdown in March 2020 forced millions of internal migrants out of the cities and back into their villages (Slater and Masih 2020). The immediate closure of the transport system meant that these people, mostly low paid workers in precarious jobs, had to walk for hundreds of miles without food or medical facilities. In Argentina, closure of internal borders between provinces, with strict requirements for special government permits to cross checkpoints, resulted in qualifying citizens unable to see dying family members (Origlia 2020) and patients dying on ambulances not allowed to enter another province (Clarin 2020). In the UK, Black and Asian communities were disproportionately hit by the pandemic, yet government regulations did little to address such disparities (Cox 2020). These examples point to a process of policymaking lacking understanding of the circumstances and needs of ordinary people, especially poor and minority groups. Greater disciplinary and identity diversity in expert advisory boards is necessary to provide decision makers with a broad spectrum of perspectives and framings on complex (wicked) social problems and their impact upon different groups so that policy options are not closed off. This ‘diversity bonus’ (Page 2017), however, may not be sufficient to bullet proof policies against social harms, because policymaking remains political and decisions are often made not because of but in spite of expert advice. Moreover, for politically charged wicked problems, decisions cannot be based on (usually uncertain) evidence alone but must entail a negotiation of values, which is arguably outside the sphere of science advice (Pielke 2007). In these situations, more open decision-making processes are needed which should bring to the table different forms of knowledge and expertise, beyond scientific expertise, as it will be argued in the last section.

## The Structural Determinants of Epistemic Narrowing

Despite lofty aspirations, diversity and inclusion remains a challenge. This is not just an issue for science advisory boards but also for many of the white-collar occupations typically associated with expertise. For example, in medicine, high occupational heritability – children following their parents’ occupations – has been suggested (Polyakova et al. 2020), meaning that those entering the profession tend to be mostly from the middle classes, thereby reducing socioeconomic diversity. Academia’s lack of diversity in terms of gender, sexuality and ethnicity is well documented, and emerging evidence suggests that socioeconomic diversity is an issue too (Morgan et al. 2021). Thus, whilst the epistemic homogeneity of advisory boards may well reflect politicised vetting processes that filter out dissenting voices (Stevens 2007), a more system-level explanation may be found in the upstream structural injustices that narrow the opportunities for certain social groups to participate in epistemic communities and contribute to knowledge production. Anderson (2012) identifies three types of structural injustices, which may be relevant here to understand the systemic causes of epistemic narrowing in science advice: *differential access to markers of credibility*, *in-group favouritism* and *shared reality bias*. Let us take each in turn.

Selection of expert advisors relies on *markers of credibility*. Education, qualifications, peer recognition and past contribution to science (as assessed through proxies like publications, and other measurable outputs), are all markers that signal the credibility of an expert and the quality of their expertise. Using these markers to establish an expert’s ability to provide sound and useful advice does not constitute per se an epistemic injustice. However, in societies where some groups systematically struggle to access opportunities to develop markers of credibility, using such markers will further exclude them from exercising epistemic agency and contributing to public life. As discussed earlier, well-documented Matthew effects in academia result in a concentration of academic prestige in privileged groups and institutions, where minorities and other disadvantaged groups tend to be underrepresented. Thus, a structural injustice – differential access to opportunities to develop successful academic careers – generates credibility deficits and leads to epistemic injustice towards the disadvantaged groups. Epistemic injustice due to unfair access to opportunities to acquire markers of credibility may lead to epistemic narrowing due to preferential selection of advisors from certain backgrounds and/or institutions.

*In-group favouritism* is a bias in favour of the group to which one belongs. Unless associated with outgroup discrimination, in-group favouritism is not unfair per se – it can foster trust and social cooperation. However, if groups are segregated along identities that are the basis of unjust inequalities, in-group favouritism may lead the dominant group to dismiss the testimony of the disadvantaged group, thus reinforcing epistemic injustice towards the latter. In-group favouritism within expert groups may not occur along lines of individual characteristics such as gender or race – although as suggested above these groups tend to be underrepresented – but epistemic injustices still can manifest along less obvious dimensions, for example along axes of competing philosophical and methodological assumptions about what constitutes good evidence. Consider for example the credibility deficit of those championing evidence obtained through experimental designs considered of ‘lower methodological quality’. The scientific orthodoxy of evidence-based medicine (EBM) translates into a ‘pecking order’ of scientific approaches, with systematic reviews and randomised-controlled trials (RCT) as the gold standard of scientific methodologies because of their ability to establish causal relationships and yield statistically significant and generalisable answers to focused questions. This creates a hierarchy of evidence, in which the use of particular (generally positivistic) methodologies determines the strength of the medical evidence. Modelled on the EBM movement, EBP communities have sought to adopt similar hierarchies to base policy decisions on sound evidence rather than on ideology, thus improving policy efficiency and effectiveness. Endorsing this approach to policymaking is not in itself unjust but favouritism towards reductionist and almost algorithmic approaches to using evidence for policy can lead to testimonial injustice against certain forms of expertise and the dismissal of valuable forms evidence.

The policy response to COVID-19 provides abundant examples of this type of in-group favouritism. Government experts favouring the knowledge of likeminded proponents of gold-standard approaches have committed testimonial injustice against those pursuing less rigid approaches to evidence generation that incorporate a variety of methodologies. As Greenhalgh (2020a, 2020b) has argued extensively, in-group favouritism led governments and their advisory boards to dismiss non-gold standard approaches and justify inaction/delayed action on the grounds of weak or absent evidence (meaning evidence from RCT and other so-called robust designs). This led to costly delays in mandating face coverings, airport testing, mass gathering bans, and other critical measures for which some (arguably non-gold standard) evidence of effectiveness already existed (Greenhalgh 2020a, 2020b). It may be argued that such delays represent instances of genuine expert disagreement. However, expert disagreement alone cannot explain reluctance to endorse measures (some indeed harmless, such as face coverings) on a precautionary principle at a time when the cost of inaction was significantly higher than action based on incomplete or uncertain evidence. Rather, in-group favouritism towards rigid adherence to the hierarchy of evidence may have led, at least in part, to the dismissal of certain experts and of evidence that fell short of the gold standard, at a time when that was the only type of evidence available.

*Shared reality bias*, also known as *groupthink* (Janis 1972), is the tendency of individuals who interact frequently to converge in their views. Again, this can be a positive feature of social groups as it reduces conflict and facilitates group cohesion and cooperation. For members of advisory groups engaged in joint epistemic activity, shared reality bias can help them present unified and coherent advice to policymakers. However, if members of advisory groups drawn from socially advantaged groups are consistently insulated from the views and perspectives of disadvantaged groups, the epistemic categories they use to make sense of social problems will be inadequate to comprehend the experiences of those from whom they are isolated. In other words, the lived reality of disadvantaged groups become unintelligible to advisors because the latter’s cognitive resources are based on the experiences they share with one another, and which are ill-suited to interpret the experiences of the former. Whilst the unequal impact of COVID-19 on social groups has been acknowledged and extensively discussed by experts, measures have consistently failed to take account of the specific circumstances of the poorest in society. From lockdown and work from home policies, to rules that enable international travel, many pandemic policies do not seem to have been designed with the poor in mind. In the UK, for example, lockdown rules allowed paid cleaners and nannies (usually servicing middle class families) into homes, but not family and friends, the vital social network upon which many low-income families depend (Bear 2020). Shared reality bias due to segregation of science advisors and policy makers from the circumstances of the poor and marginalised becomes a cause of hermeneutical injustice. Disadvantaged groups may in some cases be able to articulate coherent accounts of their experiences (consider for example Black Lives Matter protests) but such accounts fail to elicit a policy response because they are not comprehended by those in positions of advantage.

## Towards Epistemic Democracy for Postnormal Times

This paper suggests that much of the mismanagement of the COVID-19 crisis can be attributed to the failure of many governments to adopt a reflexive and inclusive approach to the governance of wicked problems, which requires engaging a diversity of knowledge and perspectives. This failure, the paper argues, is rooted in the structural epistemic injustices upon which the current system of expert advice is built. As the world emerges from the pandemic, a key question arises: *what* and *whose* knowledge can enable the development of new policy frameworks that do not discount the long-term solutions needed to build resilient societies in favour of short-term fixes? If we are to learn from the past mistakes, *how* must our current monolithic system of expertise be refashioned to ensure meaningful participation in collective processes of knowledge production and use in policymaking? The answer is not straightforward but points to the need to tackle the structural injustices that narrow the epistemological foundations of science advice and EBP.

The COVID-19 crisis is a litmus test of the epistemic injustices ingrained in knowledge systems which perpetuate the dominance of particular experts and cultures of expertise. Historic processes of epistemic narrowing may have contributed to a narrative of the pandemic as a *health* crisis. A narrow *medical gaze* (Foucault 2003, 109) sanitised the pandemic through epidemiological models and discounted the messy black box of long-term social, environmental, and economic impacts. This same gaze in the UK helped legitimise eye-watering investments in underutilised hospital infrastructure, undelivered ventilators and other underwhelming *moonshot technologies* (Deeks, Brookes, and Pollock 2020), whilst low-income families were left struggling to feed their children (Abbasi 2020).

It may be argued that while diverse expertise may enlarge the range of policy options on the table (the diversity bonus), it also risks creating a cacophony of opinions that reduces the possibilities to arrive to a shared understanding of the evidence (the diversity costs). If the costs outweigh the bonus, governments’ capacity to act may be impaired, with consequences for social justice. This, however, assumes that solving social problems hinges only or mainly on grasping some form of scientific truth. As we saw earlier, this may be true for ‘tame’ problems but not for ‘wicked’ problems. Whether to invest in ventilators or strengthen food security are decisions that cannot be based solely on scientific evidence; they require negotiation of values, paradigms, political and cultural frameworks, all of which co-exist in pluralistic societies and must come to terms with each other during the crisis and beyond. Public health crises, like most wicked problems, inhabit what Funtowicz and Ravetz (1993) call a *post-normal science* (PNS) space, navigating which necessitates broadening the knowledge base beyond scientific expertise (Rainey et al. 2021) and promoting more transparent and plural decision-making processes.

This paper has argued for epistemic justice within the institutions of knowledge creation as a necessary condition to dismantle unhelpful epistemic hierarchies and ensure diversity of expertise in science advisory bodies. However, when the science is incomplete and the expertise contested, the decision-making process requires a much deeper engagement with the values of deliberative democracy. For Funtowicz and Ravetz, such engagement takes place within extended peer communities (EPC), a socially distributed system of diversified expertise that contributes scientific and non-scientific facts to the decision-making process. Thus, alongside what we may call the *‘content expertise’* of different disciplines (cognitive diversity) and diverse expert perspectives (identity diversity), to ensure a broad evidence base we also need what we may call the *‘context expertise’* of those who experientially know about the issues at stake and feel the impact on their everyday lives. An EPC, thus, may bring scientists and policy makers together with communities, groups and individuals, whose local knowledge and contextual understanding may not be available to scientific experts and without which normative policy questions cannot be addressed (Turnpenny, Jones, and Lorenzoni 2010).

Policymakers are generally keen to create deliberative spaces where different stakeholders engage more directly in decision-making, particularly in contested policy areas, as a way to enhance policy legitimacy (Dieleman et al. 2014). Some governments have experimented with PNS approaches to decision-making, albeit with mixed results (Petersen et al. 2010). This is because it is not enough to engage stakeholders (context and content experts) in deliberative processes of policymaking. *Inclusive* does not necessarily mean *meaningful* engagement and PNS theory does not offer a solution to the question of power within the EPC (Karpińska 2018; Wesselink and Hoppe 2011). Power determines not only who participates in the EPC but also on what terms, and inviting less privileged groups to the table does not per se give them social power unless the dynamics between different groups in their struggle to influence the narrative are understood and subverted. If the answer to epistemic narrowing is a PNS type of deliberative democracy, a pre-requisite for meaningful participation is epistemic justice.

In sum, addressing the wicked problems of our times needs widened expertise in the structures and systems of knowledge creation and decision-making. To achieve this, diversifying the nature of content expertise is essential, but also valuing and meaningfully drawing on the context expertise extant in society, especially among groups typically excluded from decision-making fora. Governments should foster knowledge alliances, diverse constellations of epistemic actors coalescing around particular issues. Albeit not perfect, some spaces already exist, for example in Singapore where a programme of community conversations on issues such as ageing, cost of living and lately pandemic recovery brings together policy actors, communities and experts. Such alliances are only possible within a culture that promotes meaningful, organic dialogue beyond differences in life experiences, educational backgrounds, and identities. Such culture can be created if upstream injustices in the distribution of opportunities (and resources) to develop markers of credibility are addressed. Within communities of content experts, this calls for the much talked-up promises of widened participation within knowledge institutions (universities, think tanks, consultancies) to move beyond the empty rhetoric of equality, diversity, and inclusion that seeks to widen access to the status quo without the power shifts necessary to make structural changes. As a result, diversity initiatives end up empowering already privileged minorities whilst continuing to exclude disadvantaged and underrepresented groups. Power shifts (facilitated by more imaginative approaches to research funding) are needed also to dismantle in-group favouritism and the entrenched epistemic hierarchies that elevate positivist traditions and devalue other forms of knowledge. Shared reality biases that precludes understanding of the realities of ordinary people can be avoided if structures of science advice embrace PNS approaches and are built around EPC where content and context experts are equally valued. Pursuing epistemic democracy through inclusive and distributed deliberative post-normal spaces can foster the new approaches to EBP necessary to build preparedness and avert future crises by addressing the underlying weaknesses and vulnerabilities in our social systems. It can also help improve the systems and structures of expert advice that are needed to tackle the many wicked problems of our postnormal times.

## Conclusion

The question of what expertise (and experts) governments and society at large need in post-normal times is interrelated with the question of whether we continue to frame the COVID-19 pandemic as *the* crisis that must be overcome with the tools of science, or whether we recognise it as a wicked problem. Pandemics are not *natural* phenomena, but a biological process interweaved with a social, political and economic factors. Addressing COVID-19 now and building resilience for future shocks requires above all addressing the interrelated inequalities that are not new but have been exposed by this crisis (Mormina 2020; Smith 2021). For this, we cannot simply rely on technological solutions, but must build alternative social structures underpinned by a new social contract with deliberative democracy at its centre.

Crises are said to create the conditions for change, and history will tell to what extent the world has seized the opportunity afforded by COVID-19. This paper argues that the expertise needed at this crossroad is one that embraces greater pluralism, avoids groupthink, challenges the accepted orthodoxy, and helps us revert old models and rigid path dependencies that so often neglect the lived realities and demands of those left behind. Building back better and fairer can only be achieved by overcoming epistemic injustice and cultivating the virtue of epistemic democracy in the practices and institutions of EBP, and indeed in all social institutions.

## Notes

1. Outcomes here are defined in terms of number of COVID-related deaths per capita.

2. Essentially, growth facilitated by research income makes it more likely to subsequently succeed in securing funding, leading to further growth.

3. In addition to government appointed scientific advisors (themselves largely drawn from elite institutions), the first 20 meetings of SAGE (up to the first lockdown in March 2020) included 19 external experts, representing 10 English and one Scottish universities, as well as business, a European public health body and a not-for-profit medical research foundation. Of these, only six experts attended regularly (10 meetings or more), representing just four English Universities, all of them in the Golden Triangle.
